# Helping hands: A cluster randomised trial to evaluate the effectiveness of two different strategies for promoting hand hygiene in hospital nurses

**DOI:** 10.1186/1748-5908-6-101

**Published:** 2011-09-03

**Authors:** Anita Huis, Lisette Schoonhoven, Richard Grol, George Borm, Eddy Adang, Marlies Hulscher, Theo van Achterberg

**Affiliations:** 1 Scientific Institute for Quality of Healthcare, Radboud University Nijmegen Medical Centre Nijmegen, The Netherlands

## Abstract

**Background:**

Hand hygiene prescriptions are the most important measure in the prevention of hospital-acquired infections. Yet, compliance rates are generally below 50% of all opportunities for hand hygiene. This study aims at evaluating the short- and long-term effects of two different strategies for promoting hand hygiene in hospital nurses.

**Methods/design:**

This study is a cluster randomised controlled trial with inpatient wards as the unit of randomisation. Guidelines for hand hygiene will be implemented in this study. Two strategies will be used to improve the adherence to guidelines for hand hygiene. The state-of-the-art strategy is derived from the literature and includes education, reminders, feedback, and targeting adequate products and facilities. The extended strategy also contains activities aimed at influencing social influence in groups and enhancing leadership. The unique contribution of the extended strategy is built upon relevant behavioural science theories. The extended strategy includes all elements of the state-of-the-art strategy supplemented with gaining active commitment and initiative of ward management, modelling by informal leaders at the ward, and setting norms and targets within the team. Data will be collected at four points in time, with six-month intervals. An average of 3,000 opportunities for hand hygiene in approximately 900 nurses will be observed at each time point.

**Discussion:**

Performing and evaluating an implementation strategy that also targets the social context of teams may considerably add to the general body of knowledge in this field. Results from our study will allow us to draw conclusions on the effects of different strategies for the implementation of hand hygiene guidelines, and based on these results we will be able to define a preferred implementation strategy for hospital based nursing.

**Trial registration:**

The study is registered as a Clinical Trial in ClinicalTrials.gov, dossier number: NCT00548015.

## Background

Hospital-acquired infections (HAIs) are a serious and persistent problem throughout the world. They are burdensome to patients, complicate treatment, prolong hospital stay, increase costs, and can be life threatening [1,2].

Micro-organisms on the hands of healthcare workers contribute to the incidence of infections in patients [3,4]. Therefore, hand hygiene prescriptions are widely accepted as the most important measure in the prevention of HAIs [5-11]. Unfortunately, numerous studies over the past few decades have demonstrated that healthcare workers still perform hand hygiene on average less than 50 percent of the times required [12-14]. Thus, current practices deviate from the goal of providing safe hospital care aimed at prevention of adverse events, morbidity, and mortality.

In their review on approaches for transferring evidence to practice, Grol and Grimshaw [15] used a case study looking at strategies to improve hand hygiene in hospital settings. They concluded that plans for improvement of current performance should be based on barriers and facilitators for change. Regarding hand hygiene, they concluded that changing behaviour is possible, but this change generally requires 'a comprehensive plan with strategies at different levels (professional, team, patient, and organisation) to achieve lasting changes in hand hygiene routines.'

Traditionally, implementation strategies have focussed on professionals--the individual level--or addressed structural work context--the organisational level. Team-directed strategies are hardly studied [15,16]. Yet, team-directed strategies could be valuable as healthcare workers (especially nurses) usually work in teams. Performing and evaluating an implementation strategy that also targets the social context of teams may considerably add to the general body of knowledge in this field.

### Aims and objectives

The aim of this study is to test two implementation strategies in inpatient wards to improve nurses' compliance with hand hygiene prescriptions and to compare the short-term and sustained effects of these innovative strategies. The objectives of this project are threefold: to improve compliance with guidelines for hand hygiene in nurses; to assess the cost effectiveness of both strategies; and to gain insight into determinants of success or failure of the strategies.

### Scientific hypothesis

Our hypothesis is that an extended strategy, using additional implementation activities based on social influence and leadership, will be more effective in increasing hand hygiene compliance rates compared to a state-of-the-art strategy, mainly addressing the individual and organisational level.

## Methods

### Quality improvement strategies

The state-of-the-art strategy is based on current evidence from literature on hand hygiene compliance [1,15]. Short-term effectiveness of this strategy is well-established in several studies and settings [16,17]. The strategy includes: education for improving relevant knowledge and skills; reminders for supporting the transfer from a positive intention to the actual performance of hand hygiene; feedback as a means to provide insight into current hand hygiene behaviour and to reinforce improved behaviour; and screening for adequate hand hygiene products and adequate facilities. The extended strategy also contains activities based on social influence in groups and leadership. This strategy largely draws from relevant theories and general evidence to support these theories [18-26]. The extended strategy includes all of the above elements of the state-of-the-art strategy as well as: gaining active commitment and initiative of ward management; modelling by informal leaders at the ward; and setting norms and targets within the team. Table 1 shows the operationalisation of both strategies.

**Table 1:** Description implementation strategies

State-of-the-art strategy	Extended strategy
**Education**	**All elements of the state-of-the-art strategy**
Distribution of educational material/written information (leaflet) about hand hygiene	• Education, reminders, feedback, facilities and products
• The importance of hand hygiene	**Setting norms and targets within the team**
• Misconceptions about alcohol-based hand disinfection	• Three interactive team sessions that includes goal setting in hand hygiene performance at group level
• Theory and practical indications for the use of hand hygiene	• Analysis of barriers and facilitators to determine how they could best adapt their behaviour in order to reach their goal
Website http://www.gewoonhandenschoon.nl	• Nurses address each other in case of undesirable hand hygiene behaviour
• Educational material/written information about hand hygiene	**Gaining active commitment and initiative of ward management**
• Knowledge quiz	• Ward manager designates hand hygiene as a priority
• Reward for the nursing ward with the most visitors to the website	• Ward manager actively supports team members and informal leaders
Educational sessions on prevention of hospital acquired infections	• Ward manager discusses hand hygiene compliance rates with team members
• Launching hospital wide campaign with practical demonstrations of hand hygiene	**Modeling by informal leaders at the ward**
**Reminders**	• Informal leaders demonstrate good hand hygiene behaviour
• Distribution of posters that emphasized the importance of hand hygiene, particularly alcohol-based hand disinfection	• Informal leaders models social skills in addressing behaviour of colleagues
• Interviews and messages in newsletters or hospital magazines	• Informal leaders instruct and stimulate their colleagues in providing good hand hygiene behaviour
• General reminders by opinion leaders/ward management	
**Feedback**	
• Bar charts of hand hygiene rates of every nursing ward will be sent to the ward manager twice	
• Comparison ward performance and hospital performance	
**Facilities and products**	
• Screening and if necessary adapt products and appropriate facilities	

### Study design

The study will have a stratified cluster randomised trial design. In a cluster randomised trial, groups of individuals rather than individuals are randomised [27]. Cluster randomisation using wards as the unit of allocation reduces contamination between groups [28]. In our study, the quality improvement strategies involved the entire team of nurses and not individual nurses on nursing wards. Therefore, nurses within the same ward were considered to be a cluster.

**Figure 1:**
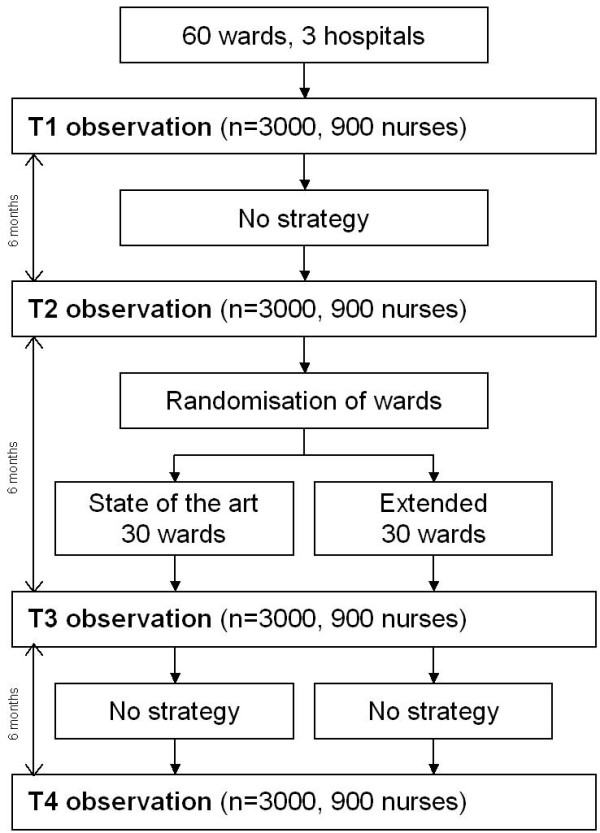


Data will be collected for a six-month reference period--no strategy for promoting hand hygiene--prior to the trial (T1 and T2). After data collection for this reference period, randomisation to either the state-of-the-art strategy or the extended strategy will take place. Strategies will be delivered during a second period of six months. Follow-up measurements will take place directly after strategy delivery (T3) and at six months after the end of strategy delivery (T4). Because the extended strategy consists of the state-of-the-art strategy supplemented with team-directed social influence approaches, randomisation of wards to each of the strategies is feasible. Our study design is illustrated in figure 1.

### Setting and participants

The study will be performed in three hospitals: one university medical centre and two general hospitals. In a fourth (non participating) hospital, we will test the instruments and observer variability. Within the hospitals, all inpatient wards (n = 60), will participate in the study.

After completing baseline measurements of the reference period, wards will be randomly assigned to either the state-of-the-art strategy group (n = 30), or the extended strategy group (n = 30). The randomisation of the wards will be stratified for type of ward to minimize differences in ward characteristics over the strategies. We will randomise surgical wards, internal medicine wards, intensive care units, and paediatric wards.

### Parameters, instruments, and analysis

To evaluate the effectiveness and efficiency of the strategies, we will use effect parameters and process parameters. First, we describe the evaluation of hand hygiene compliance and team climate. Second, the economic evaluation regarding costs and health effects. Finally, we describe the assessment of the actual implementation of the strategies and the evaluation of barriers and ward structure.

### Effect evaluation: hand hygiene compliance

Table 2 presents the effect parameters and instruments. The primary effect parameter for this study is the percentage of opportunities at which hand hygiene is performed by the nurses according to the National Guideline 'Handhygiene' of the Working group Infection Prevention (WIP) and the WHO Guidelines on Hand Hygiene in Healthcare [29,30]. The indications that create an opportunity--a required moment--for hand hygiene are listed in Table 3. Hand hygiene is operationalised as 'hand washing with either plain soap and water' or 'hand disinfection through the use of an alcohol-based hand rub solution.'

**Table 2:** Parameters and instruments

Effect parameter	Description	Instruments
Hand hygiene compliance Other parameters	The percentage of opportunities at which hand hygiene was performed according to the National Guideline 'Handhygiene' of the Working group Infection Prevention (WIP) and the WHO Guidelines on Hand Hygiene in Healthcare The percentage of presence of jewelry and long-sleeved clothes	Hand hygiene monitoring tool

Team Climate	Dimensions 'participation safety,' 'task orientation,' support for innovation,' and 'interaction.'	Team Climate Inventory

Costs and health effects	Comparing resource consumption and HAIs rate between the two implementation strategies	Activity-based costing; Decision analysis

**Process parameter**	**Description**	**Instruments**

Performance of the strategies	*State-of-the-art strategy *- Knowledge - number of nurses that completed the knowledge quiz, presence of instruction leaflets. - Reminders - check of presence of posters. - Performance feedback - actual delivery of performance feedback to team members.	Survey, direct observations; systematic registration of time and meeting minutes
	Extended strategy - Coaching of ward management- number of coaching sessions, total time spent on coaching, topics dealt with, managers evaluations of coaching. - Coaching of informal leaders - number of coaching sessions, total time spent on coaching, topics dealt with, informal leaders evaluations of coaching. - Team discussions for norm- and target setting - number of nurses attending per ward, time investment per ward, actual norms and targets decided on, nurses' evaluations of team discussions	

Barriers to change	Including determinants like awareness, knowledge, reinforcement, control, social norms, leadership, and facilities	Barrier questionnaire

Ward structure	Information about existing structures and resources like actual presence of facilities, workload, nurse-bed ratio -under-staffing and support from the management	Ward structure questionnaire

**Table 3:** Observed indications for hand hygiene

Indication for hand hygiene	When	Transmission risk	Major targeted negative infectious outcome	Examples
Before an aseptic task	Directly before performing an aseptic task	Hand transmission of micro-organisms from any surface (including the patient skin) to a site that would facilitate invasion and infection	Endogenous or exogenous infection of the patient	Giving an injection. Insertion and care of intravenous catheters. Blood draws. Administering intravenous medication. Endotracheal suction

From contaminated body site to another body site	Directly after completing task (whether gloved or ungloved)	Hand exposure to patient's contaminated body sites and fluids potentially containing blood-borne or other pathogens	Infection of the HCW by patient blood borne pathogens	Drawing blood and then adjusting the infusion drop count. Handle wound, mucous membrane, and body fluids. After oral care

After touching the patient	Directly after leaving the patient when the patient was touched	Hand transmission of micro-organisms from the patient flora to other surfaces in the healthcare setting	Dissemination of patient flora to the rest of the healthcare environment and infection of other patients or HCWs	After skin contact with the patient. Bathing, change position or lifting a patient. Taking a pulse or blood pressure. Shaking hands

After taking care of an infected/colonized patient	Directly after leaving the patient's room	Hand transmission of micro-organisms from the patient flora to other surfaces in the healthcare setting	Dissemination of patient flora to the rest of the healthcare environment and infection of other patients or HCWs	Contact with any patient know to be infectious/isolated (eg. MRSA)

After use of gloves	Directly after removing gloves	Hand transmission of micro-organisms from the skin of the HCW 's to other surfaces in the healthcare setting	Dissemination of patient flora to the rest of the healthcare environment and infection of other patients or HCWs	Wearing gloves high-risk contacts

After contact with patient surroundings	After completing the task and before contacting another patient	Hand transmission of micro-organisms from the patient flora to other surfaces in the healthcare setting	Dissemination of patient flora to the rest of the healthcare environment and infection of other patient or HCWs	Touching the patient's environment like bed, table, blanket, clothes. After contact with medical equipment in the immediate vicinity of the patient

Other effect parameters are the presence of jewelry (ring, watch, or other jewelry) and whether the nurses wear long-sleeved clothes under their short-sleeved uniforms. We will observe compliance by using a Hand Hygiene Monitoring Tool adapted from the WHO (additional file 1). The observer will register each opportunity in a corresponding column block, note all of the applicable indications and whether hand hygiene is performed by hand disinfection or hand washing or is missed.

### Data collection

At each point in time, an average of 3,000 opportunities for hand hygiene in approximately 900 nurses will be observed. We will use direct, but unobtrusive observation because this is considered the gold standard and the most reliable method for assessing compliance rates [1,31-33]. At the beginning of each observation period, nurses will be informed that the observers are conducting research on medication errors and other patient safety issues, but not that hand hygiene will be monitored. Observers will conduct their observations at times with a high density of care, mostly during the morning shifts. Observers will be blinded for the strategies delivered to the wards under observation.

### Observer variability

For each observation period, we will train 10 student nurses, all completing their nursing education and experienced in patient care, as well in collecting data. All student nurses will participate in a two-day training course on understanding the indications for hand hygiene during patient care. They will also learn to apply the observation method and to use the data collection form. Before conducting the observation sessions, the observations by the student nurses will be validated. Visual examples of patient care episodes will be presented, and the students will score related hand hygiene opportunities. Then, we will compare the results of the students and discus discordant notifications. Subsequently, we will undertake parallel monitoring sessions in a non-participating hospital. Every student nurse will perform twenty observations jointly with an experienced observer.

We will use a three-step approach to compare the concordance between the observer and the experienced observer. First, we will calculate the concordance between 'the number of recorded hand hygiene opportunities' of the student nurse and the experienced observer. Then, we will calculate the concordance between 'the number of recorded hand hygiene indications' of both observers. Finally, we will calculate the concordance between 'the number of recorded actions.' The Wilcoxon rank test will be used to detect differences between the student nurses and experienced observer.

### Statistical analysis

The effects of the two strategies will be evaluated on an intention-to-treat basis by comparing the hand hygiene compliance rates in the two study groups after performing the strategies with the compliance rates at the end of the reference period. The differences between the two strategies will be evaluated by comparing the hand hygiene compliance rates of both groups after performing the strategies. Multilevel analysis will be applied to compensate for the clustered nature of the data (compliance is clustered within healthcare workers who are clustered within units) using mixed linear modelling techniques, including the following covariates: ward (random effect), HCW (random effect, nested within ward), institution and the baseline results of the wards. The relevance of nurses' gender, ward specialism, and type of hand hygiene opportunity will also be explored by performing sub group analyses.

### Sample size

The state-of-the-art implementation strategy should be able to improve hand hygiene compliance with 15% in the short term [1]. We assume an added effect of 10% from the team-directed approach. This means that the extended strategy would be clinically relevant if it would result in an improvement of compliance with 25% of all occasions for hand hygiene. Calculating from 80% power, two-sided alpha = 0.05, a ward-ICC of 0.05 and a nurse-ICC of 0.6, in each of the 60 wards in the study an average of 50 observations of occasions for hand hygiene compliance are needed at each point in time, involving 15 nurses per ward.

### Effect evaluation: team climate

As the extended strategy will target social interaction in teams of nurses, it is assumed that team climate will be affected in wards receiving this strategy, and not in wards receiving the state-of-the-art strategy. Team climate will be assessed at T2 and T3, in half of the nurses from each ward. For this purpose, the Team Climate Inventory (TCI) will be used [34]. The TCI includes 44 items on the dimensions 'participation safety,' 'task orientation,' support for innovation' and 'interaction.'

### Economic evaluation: costs and health effects

Costs of infections are high, and hand hygiene is a proven effective measure in reducing infections. Therefore, strategies that focus on and result in increasing compliance to hand hygiene guidelines are likely to be cost-effective. The economic evaluation will compare the two implementation strategies as described earlier in this paper both in terms of implementation costs and health effects. The aim of this evaluation is to detect which of the implementation strategies is the most cost-effective strategy for improving hand hygiene compliance and reducing HAIs. This results in two incremental cost-effectiveness ratios--cost per percentage gained compliance and cost per percentage HAI prevented.

### Data collection

The resources consumed by the implementation strategies will be assessed by collecting data on personnel (hours for the strategy delivery team, hours for the nurses attending the strategy related activities, extra time for hand hygiene), and materials (posters, improved products and facilities, use of hand-rub solution). These volumes will be multiplied by their unit prices (market prices, guideline prices or self-determined prices based on costing methods, *i.e.*, full costing [35]. The cost estimate for a hospital acquired infection and additional healthcare costs will be based on previous estimates of €4386 euro per infection [36].

### Statistical analysis

The implementation process and consequent costs will be estimated by an Activity Based Costing (ABC) approach. The ABC model focuses on identifying all the underlying activities (personnel, material and overhead costs) associated with the state-of-the-art strategy and the extended strategy.

The health effects of the implementation strategies for reducing hospital-acquired infections will be analyzed using decision analysis. We assume a baseline prevalence of infection of 6.6%, based on the data from The PREZIES national network for the surveillance of HAIs in The Netherlands [37]. With regard to the association between infection rates and hand hygiene compliance rates, a pooled (if possible) estimation will be applied. For this purpose, we will perform a review of the literature, using systematic review methodology, to identify studies that report of the impact of hand hygiene on HAIs. Studies should at least include outcome comparison with a (randomized or non randomized) comparison group, or a comparison with baseline data in case of a single group pre-test post-test design. Studies will be further selected if they satisfy the following conditions:

1. Population: healthcare workers in hospital settings.

2. Intervention: strategies or programmes aimed at improving hand hygiene behaviour.

3. Comparison: hand hygiene behaviour and infection rates.

a. Hand hygiene behaviour prior to the introduction of the program or strategy.

b. Infection rates in health-care settings prior to the introduction of the program or strategy.

4. Outcome: hand hygiene behaviour and infection rates.

a. All operationalisations of hand hygiene behaviour in healthcare workers.

b. Infection rates in healthcare setting.

### Systematic evaluation of implementation fidelity

In trials on the effects of implementation strategies, a process evaluation can shed light on the target group members' actual exposure to the strategy [38]. In this manner, insight is gained into potential determinants of success or failure of the strategies. This step also will aid in replicating the strategy in future research. For this purpose, process data will be gathered for each of the activities within the state-of-the-art strategy and the extended strategy.

### State-of-the-art strategy

Participation in education will be assessed by measuring the number of nurses that completed the knowledge quiz and by monitoring the presence of instruction leaflets on the ward. Use of reminders will be checked by measuring the presence of reminders (posters) at random moments during the strategy delivery period. Whether performance feedback was provided will be assessed by measuring the extent to which the ward manager provided feedback to the nurses. In addition, the extent to which products and facilities were available will be checked by measuring the presence of products and facilities in each ward.

### Extended strategy

The use of coaching of either ward management or informal leaders will be assessed by measuring the number of coaching sessions, the total time spent on coaching, and the topics covered during the session. The use of organised team discussions for norm and target setting will be checked by measuring the number of team discussions performed, the number of nurses attending per ward, the time investment per ward, and the actual norms and targets decided on. Process evaluation data will be collected using a combination of data-collection methods, including questionnaires, direct observations, and systematic registration of time and meeting minutes. For each of the elements of the strategies 'actual exposure' to the strategy element at the level of wards will be coded as 'low,' 'moderate' or 'high' based on the process indicator data collection. Relations between strategy exposure and hand hygiene compliance after the delivery of the strategies will be explored.

### Evaluation of barriers and ward structure

Previous recommendations from literature have pointed out that an improvement strategy for hand hygiene behaviour should address existing problems and barriers [21,39,40]. Grol and Grimshaw studied the failing implementation of evidence on hand hygiene in the healthcare setting and identified a variety of barriers to change, including a lack of awareness, knowledge, reinforcement, control, social norms, leadership, and facilities [15]. In our study, these identified barriers to change will be targeted by either the state-of-the-art strategy or the extended strategy. The presence of barriers will be investigated twice--before and after strategy delivery--using a questionnaire in one-half of the nurses from each ward. The barrier questionnaire contains 47 different propositions concerning 21 barriers.

To collect information about existing structures and resources, such as actual presence of facilities, workload, nurse-bed ratio, understaffing, and support from the management, a questionnaire on ward structure will be administered twice to every ward manager.

### Ethical and legal aspects

The Medical Ethics Committee of district Arnhem-Nijmegen assessed the study and concluded that our study was deemed exempt from their approval because it did not include collection of data at the level of patients.

The Hawthorne effect is probably the most important bias in hand hygiene observations [1,30,33,41]. Persons who know they are being observed change their behaviour and are significantly more likely to wash or disinfect their hands. Unobtrusive observation diminishes the Hawthorne effect, but raises ethical questions regarding privacy of the observed participants. Therefore, we consulted the ethical committee. They concluded that unobtrusive observation will be permitted under the following conditions: the observation topic, hand hygiene, will be covered by using general patient safety issues as subject of the observation; the observations on the nurses should be collected and processed anonymously; and prior to the observation, the patient has given verbal permission to observe.

## Discussion

Changes in healthcare can target individual professionals, teams and units, or healthcare organisations [15]. Traditionally, implementation strategies are directed at individual professionals (individual level) or address structural work context (organisational level), whereas team-directed strategies are rarely studied. The unique contribution of the extended strategy was built upon social learning theory, Social influence theory [23], theory on team effectiveness [20,20,25,25,26,26] and leadership theory [24]. Together, these theories provide a coherent set of methods to target the social context in which hand hygiene behaviour takes place. Because targeting social context is not often employed in implementation strategies, the results of our project will considerably add to the general body of knowledge by evaluation of the added value of the extended strategy as compared to the state-of-the-art strategy.

Results from our study will allow us to draw conclusions on the effects of different strategies for the implementation of hand hygiene guidelines, and based on these results we will be able to define a preferred implementation strategy for hospital-based nursing. Our evaluation of the state-of-the-art strategy will validate the effectiveness of this strategy in Dutch hospital care. The evaluation will further provide a longer term follow-up effect estimate, whereas commonly only effects during or directly after strategy delivery are evaluated [15,16].

We believe our study has methodological strengths because of the large numbers of observations and participating wards, the randomisation of wards either to the state-of-the-art strategy or the extended strategy, and the use of unobtrusive observations.

We anticipate several challenges in conducting this study. First, in an ideal world, one would choose randomisation of wards or teams to three groups: a state-of-the-art strategy group, an extended strategy group, and a no strategy group. However, as the state-of-the-art strategy includes hospital-wide campaign elements (*e.g*., posters on doors, instruction leaflets, and short articles in hospital magazines), three-group randomisation at the level of wards would certainly introduce contamination of the no strategy group. This implies that three-group randomisation in the same hospital is not a feasible option. We will collect baseline data twice, with a six month interval, in order to create a reference period with no strategy. Second, timely and accurate data collection for this study is also challenging. To ensure that comprehensive data collection is feasible in all participating hospitals, we will partner with an established Faculty of Health and Social Studies in recruiting, training, and assessing the students who will perform the observations.

Third, in this study we will not measure nosocomial infections. Measuring nosocomial infections on ward level and correcting for all possible interference from other factors would be labour intensive and costly. Given the fact that the relationship between hand hygiene and the occurrence of infections already is well established, and given practical difficulties in achieving comparable patient groups with regard to risk factor and scoring patients who transfer between wards, we decided to use a model-based estimate of HAIs.

Finally, we will not measure compliance in physicians or other healthcare workers. The main reason for not including physicians is the difference in team structure and teamwork between nurses and physicians. Whereas hospital nurses typically work and interact in ward-based teams, physicians more often work independently and on various locations. Targeting physician-directed social influence would ask for strategies other than targeting nurse-directed social influence. Nevertheless, the state-of-the-art strategy is visible to all hospital staff, and may affect physicians' hand hygiene as well.

We believe that by performing this study, we will improve hand hygiene behaviour and contribute to the body of knowledge on effective strategies for implementing hand hygiene guidelines in healthcare settings. We will specifically add knowledge to the social influence based implementation activities.

## Competing interests

The authors declare that they have no competing interests.

## Source of funding

This study is funded by a research grant from ZonMw, dossier number: 94517101.

## Authors' contributions

TVA, LS, and MH were responsible for the research question and designed the study. RG, EA, and GB commented on the design. AH wrote the first draft of this manuscript and was responsible for the revisions. TVA, LS, RG, and MH contributed to drafting of the manuscript. GB is the statistician and performed the power calculation, the sample size considerations, and offered advice on the statistical analysis. EA is the team's expert in economic evaluations and was involved in the design of the study. TVA is the general supervisor of the study and was involved in revising the article. All authors read and approved the final version of the manuscript.

## Supplementary Material

Additional file 1 **Hand Hygiene Monitoring Tool**. Scoreform Hand Hygiene opportunities. Click here for file

## References

[B1] Pittet D Hugonnet S Harbarth S Mourouga P Sauvan V Touveneau S Effectiveness of a hospital-wide programme to improve compliance with hand hygiene. Infection Control Programme Lancet 2000 356 1307 1312 10.1016/S0140-6736(00)02814-2 11073019

[B2] World Health Organization The first Global Patient Safety Challenge: Clean Care is Safer Care http://www.who.int/gpsc/en/23805438

[B3] Lucet JC Rigaud MP Mentre F Kassis N Deblangy C Andremont A Hand contamination before and after different hand hygiene techniques: a randomized clinical trial J Hosp Infect 2002 50 276 280 10.1053/jhin.2002.1202 12014900

[B4] Pittet D Dharan S Touveneau S Sauvan V Perneger TV Bacterial contamination of the hands of hospital staff during routine patient care Arch Intern Med 1999 159 821 826 10.1001/archinte.159.8.821 10219927

[B5] Day M Chief medical officer names hand hygiene and organ donation as public health priorities BMJ 2007 335 113 17641313 10.1136/bmj.39280.523657.4E PMC1925172

[B6] Donaldson L Dirty hands...the human cost 2006 London: UK Department of public health

[B7] Larson E A causal link between handwashing and risk of infection? Examination of the evidence Infect Control Hosp Epidemiol 1988 9 28 36 10.1086/645729 19722934

[B8] Larson EL APIC guideline for handwashing and hand antisepsis in healthcare settings Am J Infect Control 1995 23 251 269 10.1016/0196-6553(95)90070-5 7503437

[B9] Lautenbach E Shojania K, Duncan B, MacDonald K, Wachter R Practices to Improve Handwashing Compliance Making healthcare safer: a critical analysis of patient safety practices 2001 Rockville, MD: Agency for Healthcare Research and Quality 125 131

[B10] Pittet D Allegranzi B Sax H Dharan S Pessoa-Silva CL Donaldson L Evidence-based model for hand transmission during patient care and the role of improved practices Lancet Infect Dis 2006 6 641 652 10.1016/S1473-3099(06)70600-4 17008173

[B11] Teare E Cookson B French G Hand washing--a modest measure with big effects BMJ 1999 318 68610073995

[B12] Kuzu N Ozer F Aydemir S Yalcin AN Zencir M Compliance with hand hygiene and glove use in a university-affiliated hospital Infect Control Hosp Epidemiol 2005 26 312 315 10.1086/502545 15796286

[B13] Pittet D Mourouga P Perneger TV Compliance with handwashing in a teaching hospital. Infection Control Program Ann Intern Med 1999 130 126 130 10068358 10.7326/0003-4819-130-2-199901190-00006

[B14] Pittet D Improving compliance with hand hygiene in hospitals Infect Control Hosp Epidemiol 2000 21 381 386 10.1086/501777 10879568

[B15] Grol R Grimshaw J From best evidence to best practice: effective implementation of change in patients' care Lancet 2003 362 1225 1230 10.1016/S0140-6736(03)14546-1 14568747

[B16] Naikoba S Hayward A The effectiveness of interventions aimed at increasing handwashing in healthcare workers - a systematic review J Hosp Infect 2001 47 173 180 10.1053/jhin.2000.0882 11247676

[B17] Grimshaw J Eccles M Thomas R MacLennan G Ramsay C Fraser C Toward evidence-based quality improvement. Evidence (and its limitations) of the effectiveness of guideline dissemination and implementation strategies 1966-1998 J Gen Intern Med 2006 21 Suppl 2 S14 S20 16637955 10.1111/j.1525-1497.2006.00357.x PMC2557130

[B18] Bandura A Social foundation and thought of action: a social cognitive theory 1986 New York: Prentice Hall

[B19] Bosch M Faber MJ Cruijsberg J Voerman GE Leatherman S Grol RP Review article: Effectiveness of patient care teams and the role of clinical expertise and coordination: a literature review Med Care Res Rev 2009 66 5S 35S 10.1177/1077558709343295 19692553

[B20] Firth-Cozens J Celebrating teamwork Qual Healthcare 1998 7 Suppl S3 S7 10339032

[B21] Grol R Wensing M Grol R, Wensing M, Eccles M Effective implementation: A model Improving patient care: The implementation of change in clinical practice 2005 London: Elsevier 41 57

[B22] Larson EL Early E Cloonan P Sugrue S Parides M An organizational climate intervention associated with increased handwashing and decreased nosocomial infections Behav Med 2000 26 14 22 10.1080/08964280009595749 10971880

[B23] Mittman BS Tonesk X Jacobson PD Implementing clinical practice guidelines: social influence strategies and practitioner behavior change QRB Qual Rev Bull 1992 18 413 422 1287523 10.1016/s0097-5990(16)30567-x

[B24] Øvretveit J The Leaders' Role in Quality and Safety Improvement; a review of Re-search and Guidance; the 'Improving Improvement Action Evaluation Project 2004 Stockholm: Association of County Councils (Lanstingsforbundet)21892042

[B25] Shortell SM Marsteller JA Lin M Pearson ML Wu SY Mendel P The role of perceived team effectiveness in improving chronic illness care Med Care 2004 42 1040 1048 10.1097/00005650-200411000-00002 15586830

[B26] West MA West MA, FJl The social psychology of innovation in groups Innovation and creativity at work: Psychological and Organizational Strategies 1990 Chichester: John Wiley and Sons 309 333

[B27] Campbell MC Elbourne D Altman D CONSORT statement: extension to cluster randomised trials BMJ 2004 328 702 708 10.1136/bmj.328.7441.702 15031246 PMC381234

[B28] Eccles M Grimshaw J Campbell M Ramsay C Research designs for studies evaluating the effectiveness of change and improvement strategies Qual Saf Healthcare 2003 12 47 52 10.1136/qhc.12.1.47 PMC1743658 12571345

[B29] Werkgroep Infectiepreventie: Handhygiëne medewerkers ziekenhuizen http://www.wip.nl/free_content/Richtlijnen/Handhygiene_medewerkers_071015def.pdf

[B30] World Health Organization WHO Guidelines on Hand Hygiene in Healthcare First Global Patient Safety Challenge: Clean Care is Safer Care http://whqlibdoc.who.int/publications/2009/9789241597906_eng.pdf 23805438

[B31] Boyce JM Hand hygiene compliance monitoring: current perspectives from the USA J Hosp Infect 2008 70 Suppl 1 2 7 18994674 10.1016/S0195-6701(08)60003-1

[B32] Braun BI Kusek L Larson E Measuring adherence to hand hygiene guidelines: a field survey for examples of effective practices Am J Infect Control 2009 37 282 288 10.1016/j.ajic.2008.09.002 19118921

[B33] Sax H Allegranzi B Chraiti MN Boyce J Larson E Pittet D The World Health Organization hand hygiene observation method Am J Infect Control 2009 37 827 834 10.1016/j.ajic.2009.07.003 20004812

[B34] Anderson NR WM Measuring climate for work group innovation: development and variability of the team climate inventory Journal of Organizational Behavior 1998 19 235 258 10.1002/(SICI)1099-1379(199805)19:3<235::AID-JOB837>3.0.CO;2-C

[B35] Oostenbrink J Bouwmans C Koopmanschap M Rutten F Handleiding voor kostenonderzoek, methoden en standaard kostprijzen voor economische evaluaties in de gezondheidszorg 2005 Diemen: College voor zorgverzekeringen

[B36] Plowman R Graves N Griffin M Roberts J Swan T Cookson B The Socio-economic burden of hospital acquired infection 1999 London: Public Health Laboratory Service

[B37] Benthem van B Kooi van der T Hopmans T Wille J Trend in prevalentie van ziekenhuisinfecties in Nederland 2007-2009 Infectieziekten Bulletin 2010 21 226 229

[B38] Hulscher ME Laurant MG Grol RP Process evaluation on quality improvement interventions Qual Saf Healthcare 2003 12 40 46 10.1136/qhc.12.1.40 PMC1743654 12571344

[B39] Bosch M van der WT Wensing M Grol R Tailoring quality improvement interventions to identified barriers: a multiple case analysis J Eval Clin Pract 2007 13 161 168 10.1111/j.1365-2753.2006.00660.x 17378860

[B40] Grol R Wensing M What drives change? Barriers to and incentives for achieving evidence-based practice Med J Aust 2004 180 S57 S60 15012583 10.5694/j.1326-5377.2004.tb05948.x

[B41] Eckmanns T Bessert J Behnke M Gastmeier P Ruden H Compliance with antiseptic hand rub use in intensive care units: the Hawthorne effect Infect Control Hosp Epidemiol 2006 27 931 934 10.1086/507294 16941318

