# Supplementary figures and images for: Helping hands: A cluster randomised trial to evaluate the effectiveness of two different strategies for promoting hand hygiene in hospital nurses
Source: Implement Sci. 2011 Sep 3;6:101. doi: 10.1186/1748-5908-6-101 (PMC3177889; doi:10.1186/1748-5908-6-101)

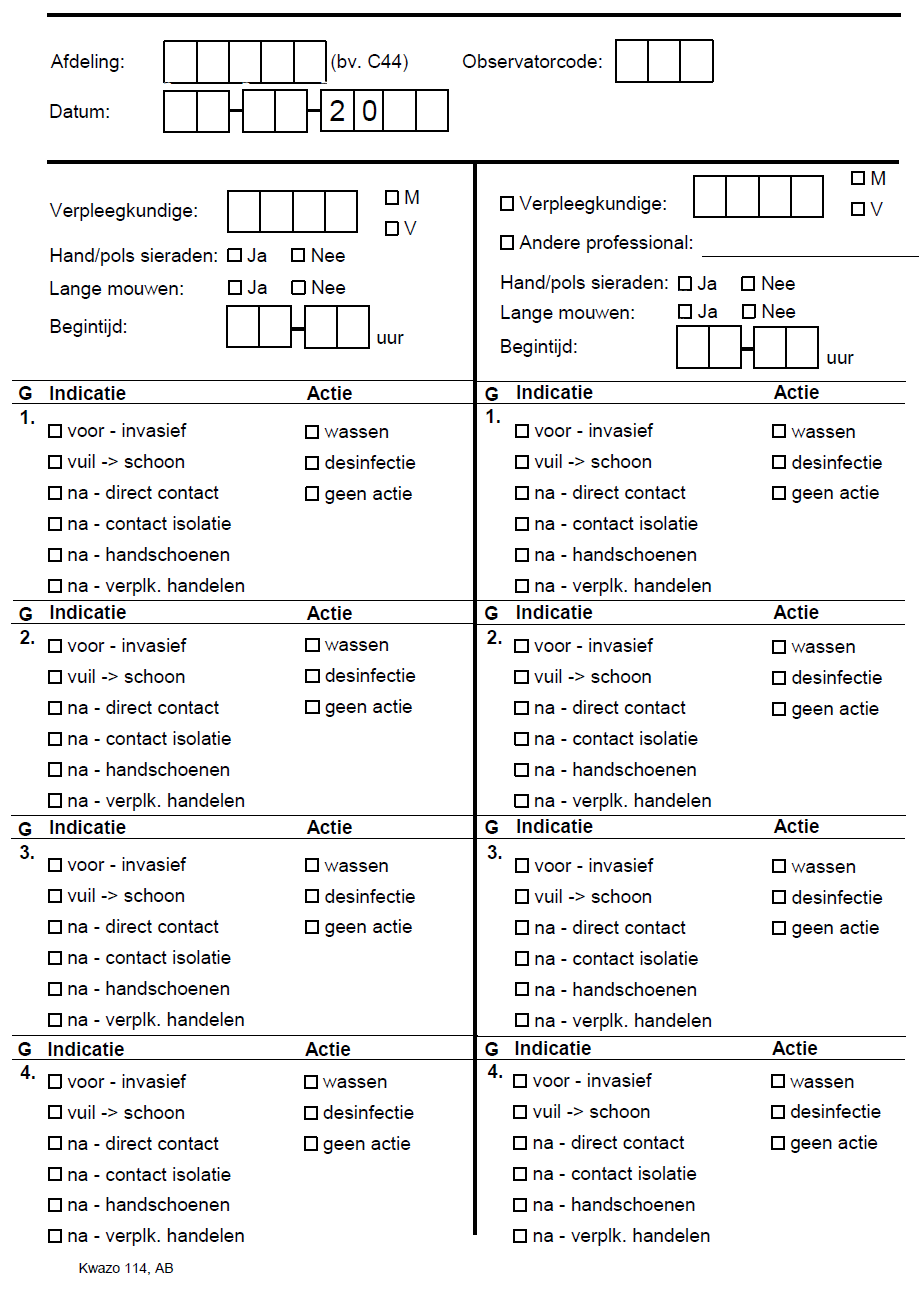

Supplement: Additional file 1 — Hand Hygiene Monitoring Tool. Scoreform Hand Hygiene opportunities. [file 1748-5908-6-101-S1.BMP]
